# Implementation of sodium-glucose co-transporter 2 inhibitors use in patients with heart failure across the ejection fraction: data from the Swedish heart failure registry

**DOI:** 10.1093/ehjqcco/qcag063

**Published:** 2026-04-19

**Authors:** Giulia Ferrannini, Francesca Musella, Lina Benson, Valeria Valente, Felix Lindberg, Marco Boccalatte, Carin Corovic Cabrera, Ulf Dahlström, Lars H Lund, Davide Stolfo, Gianluigi Savarese

**Affiliations:** Cardiology Unit, Department of Medicine Solna, Karolinska Institutet, Eugeniavägen 27, 111 11 Stockholm, Sweden; Internal Medicine Unit, Södertälje Hospital, Rosenborgsgatan 10, 152 40 Södertälje, Sweden; Department of Clinical Science and Education, Södersjukhuset, Karolinska Institute, Sjukhusbacken 10, 118 83 Stockholm, Sweden; Cardiology Department, Santa Maria Delle Grazie Hospital, Via Domiziana, Localitá La Schiana, 80078 Pozzuoli, Naples, Italy; Cardiology Unit, Department of Medicine Solna, Karolinska Institutet, Eugeniavägen 27, 111 11 Stockholm, Sweden; Department of Clinical Science and Education, Södersjukhuset, Karolinska Institute, Sjukhusbacken 10, 118 83 Stockholm, Sweden; Department of Clinical Science and Education, Södersjukhuset, Karolinska Institute, Sjukhusbacken 10, 118 83 Stockholm, Sweden; Department of Clinical Science and Education, Södersjukhuset, Karolinska Institute, Sjukhusbacken 10, 118 83 Stockholm, Sweden; Cardiology Department, Santa Maria Delle Grazie Hospital, Via Domiziana, Localitá La Schiana, 80078 Pozzuoli, Naples, Italy; Department of Clinical Science and Education, Södersjukhuset, Karolinska Institute, Sjukhusbacken 10, 118 83 Stockholm, Sweden; Department of Health, Medicine and Caring Sciences Linköping University, Lasarettsgatan 12, 582 25 Linköping, Sweden; Cardiology Unit, Department of Medicine Solna, Karolinska Institutet, Eugeniavägen 27, 111 11 Stockholm, Sweden; Department of Clinical Science and Education, Södersjukhuset, Karolinska Institute, Sjukhusbacken 10, 118 83 Stockholm, Sweden; Cardiothoracic Department, Azienda Sanitaria Universitaria Friuli Centrale and University Hospital of Udine, via Pozzuolo 330, 33100 Udine, Italy; Department of Clinical Science and Education, Södersjukhuset, Karolinska Institute, Sjukhusbacken 10, 118 83 Stockholm, Sweden

**Keywords:** Sodium-glucose co-transporter 2 inhibitors, Heart failure, Ejection fraction, Implementation, Registry, SwedeHF

## Abstract

**Aims:**

The aim of this study was to assess the implementation of sodium–glucose co-transporter-2 inhibitors (SGLT2i) use in a real-world heart failure (HF) cohort.

**Methods and results:**

Patients from the Swedish HF Registry enrolled 1 September 2022 to 16 December 2024 were included. Characteristics independently associated with SGLT2i use were investigated by multivariable logistic regression models, in the overall cohort and stratified by ejection fraction (EF).

Of 24 578 patients (median age [Q1–Q3]:75 [65–81] years, 33% female), 43% had HF with reduced EF(HFrEF), 31% had HF with mildly reduced EF(HFmrEF), and 26% had HF with preserved EF(HFpEF). During the study period, SGLT2i use increased from 78% to 89% in HFrEF, from 54% to 80% in HFmrEF, and from 41% to 80% in HFpEF.

Independent predictors of SGLT2i use included male sex, HF hospitalization during the past year, impaired kidney function, obesity, higher socioeconomic profile, and beta-blocker use. Follow-up in specialist care was associated with higher use in HFrEF and HFmrEF. Type 2 diabetes (T2DM) was independently associated with use across EF, with a stronger association in HFpEF. Renin–angiotensin–aldosterone system inhibitors were associated with SGLT2i use across the EF spectrum, with stronger associations in HFrEF and HFmrEF, while mineralocorticoid receptor antagonists showed similar associations across EF categories, stronger in HFrEF and HFmrEF.

**Conclusion:**

SGLT2i were rapidly implemented in clinical practice, with their use reaching ∼90% in HFrEF and approaching a plateau at ∼80% in HFmrEF and HFpEF in December 2024. There is still room for treatment optimization, particularly among women, patients with HFpEF without T2DM, those followed in primary care, and with lower socioeconomic status.

## Graphical Abstract

**Graphical Abstract:**
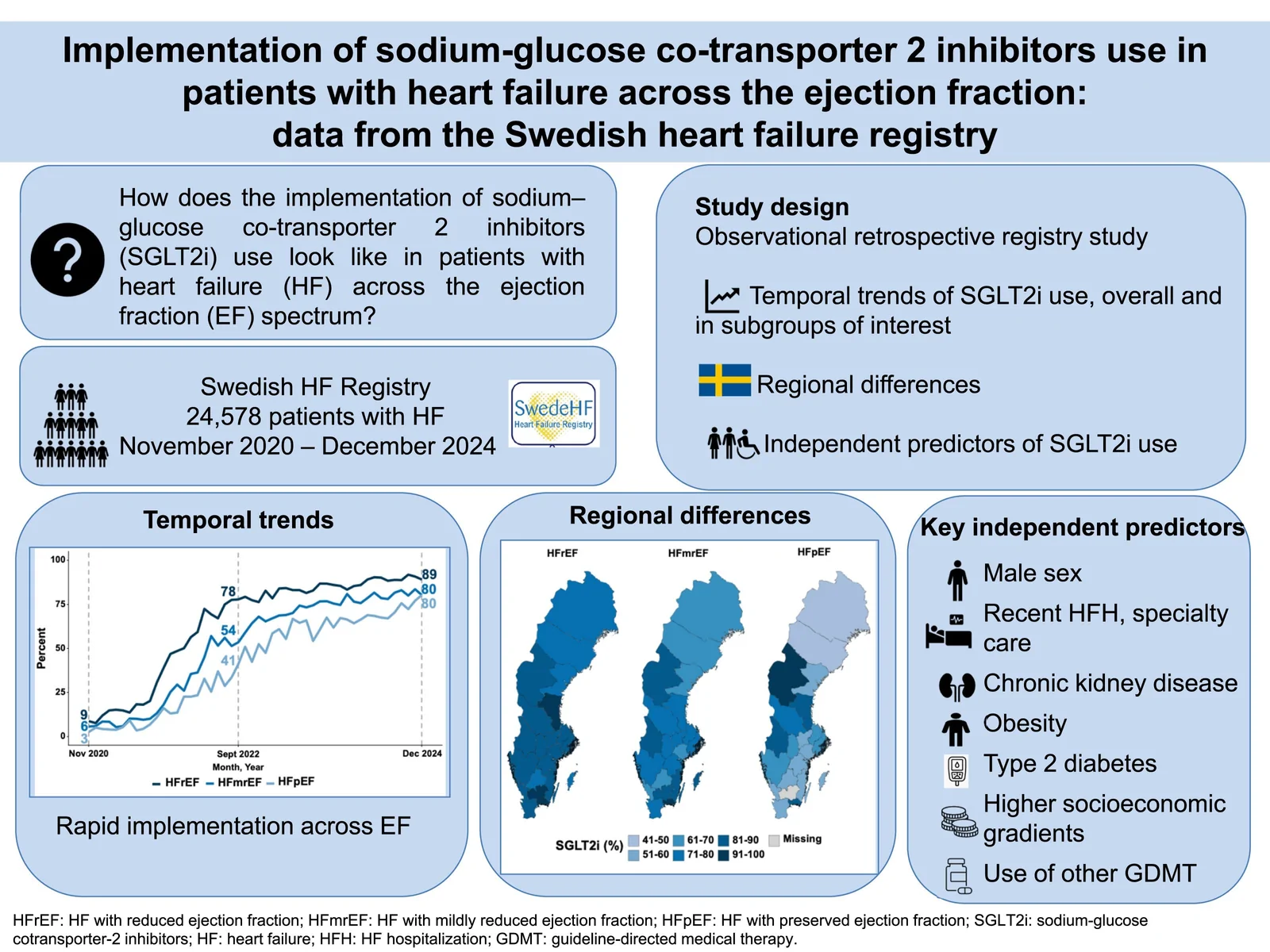

Key Learning Points What is already known:

- Sodium-glucose cotransporter-2 inhibitors (SGLT2i) in heart failure (HF) are the only guideline-directed medical therapy with class IA recommendation across the ejection fraction (EF) spectrum.
- It is known that the implementation of these drugs in HF with reduced EF (HFrEF) has been facilitated by their tolerability, safety and no need for dose titration. Less is known about implementation in patients with mildly reduced or preserved EF (HFmrEF and HFpEF).

What this study adds:

- This study, conducted on HF-specific registry data in Sweden, reports that SGLT2i implementation was remarkably fast in HFmrEF and HFpEF as well.
- Nonetheless, there are still subgroups of patients where the use of these drugs can be better implemented: In female patients, older individuals, and those with lower socioeconomic status. We also report significant regional variations and higher use in patients followed in specialist care.
- This study highlights possible obstacles to overcome as regards SGLT2i use, to help clinicians fill the gaps and guarantee the use of these highly effective drugs in all HF patients without contraindications.

## Introduction

Heart failure (HF) is a chronic syndrome characterized by high morbidity and mortality, substantial impact on quality of life, and heavy burden on healthcare expenditures.^1^ Sodium-glucose cotransporter-2 inhibitors (SGLT2i), namely dapagliflozin, empagliflozin and sotagliflozin, reduce morbidity and mortality and improve quality of life in patients with HF across the whole ejection fraction (EF) spectrum, representing so far the only guideline-directed medical therapy (GDMT) with class IA recommendation not only in HF with reduced (HFrEF), but also mildly reduced (HFmrEF) and preserved EF (HFpEF) according to the European Society of Cardiology (ESC) Guidelines on HF.^2–7^ In HFrEF patients, where they obtained guideline recommendations first, the implementation of these drugs has been facilitated by their tolerability, safety and absence of need for dose titration.^8,9^ However, the different profile of HFpEF patients, characterized by older age, different sex distribution and setting of care, presence of multimorbidity and correlated polypharmacy, frailty, as well as less awareness of the disease, might hinder SGLT2i in this EF subtype adoption in daily clinical practice.^10–12^

Whether the implementation of SGLT2i is as rapid in HFpEF and HFmrEF as it has been in HFrEF, and whether the determinants of use differ across EF phenotypes, remains to be fully elucidated. A previous analysis from the Swedish Heart Failure Registry (SwedeHF) focused exclusively on patients with HFrEF and described early uptake patterns after initial approval.^9^ However, the landscape has substantially evolved following the publication of the DELIVER and EMPEROR-Preserved trials, and the 2023 ESC focused update of the 2021 ESC Guidelines on HF granted a Class IA recommendation for SGLT2i across the EF spectrum.^7^

Therefore, the present study aims to provide an updated and comprehensive evaluation of SGLT2i implementation in a nationwide real-world HF population, with particular focus on HFmrEF and HFpEF, examining EF-specific determinants of use, and exploring socioeconomic and regional patterns of implementation during a period of rapidly expanding indications.

## Methods

### Data sources

The SwedeHF is an ongoing nationwide quality registry started in 2000, which includes in- and out-patients with HF, regardless of EF and previous history of HF.^13^ Around 80 variables are recorded at discharge from the hospital or at the outpatient visit and entered in an electronic database managed by the Uppsala Clinical Research Center (Uppsala, Sweden). Up to April 2017, the only inclusion criterion was a clinical diagnosis of HF, which was thereafter defined according to the ICD-10 codes I50.0, I50.1, I50.9, I42.0, I42.6, I42.7, I25.5, I11.0, I13.0, I13.2. In 2023, SwedeHF had a 59% coverage of the prevalent HF population in Sweden.^14^

For the current analysis, SwedeHF was linked with the following national Swedish registries, administered by the National Board of Health and Welfare, through the Swedish personal identity number: the Patient Register for obtaining additional comorbidities; the Cause of Death Register for gaining information on vital status and cause of death; and the Prescribed Drug Register for obtaining data on SGLT2i dispensations. Linkage with the Longitudinal Integrated Database for health insurance and labour market studies (LISA) and the Register of the Total Population (Statistics Sweden) were also performed to retrieve information on socioeconomic factors.

### Study population

Adult patients registered in SwedeHF between 1 September 2022 and 16 December 2024 were included. The index date was the date of patient’s registration in SwedeHF, corresponding to either the date of the outpatient visit for outpatients or the date of discharge for inpatients. In case a patient was registered more than once during the study period, the last registration only was selected, to eliminate the need to account for within-patient correlation in case of multiple registrations. For the analyses on temporal trends of use, we included patients from 1 November 2020, as this date marked the approval of SGLT2i for treatment of HFrEF in Sweden. A patient was considered as receiving SGLT2i if a dispensation was recorded in the National Prescribed Drug Registry during the 4 months before or 14 days after the index date; thus, patients without at least 14 days of follow-up were also excluded. We excluded patients who died during the hospitalization that prompted the enrolment in the registry. Key exclusion criteria were missing data for EF value and/or estimated glomerular filtration rate (eGFR), presence of type 1 diabetes or unknown diabetes type and eGFR <25 mL/min/1.73 m^2^. Only empagliflozin and dapagliflozin (alone or in combination with other glucose-lowering drugs) were considered, as they are the only SGLT2i with guidelines recommendations for use in chronic HF.

Detailed flowchart and inclusion/exclusion criteria are reported in Supplementary material online, *Table S1*. Variable definitions are reported in Supplementary material online, *Table S2* and additional information on data sources, definitions, etc., can be found at https://kiheartfailure.github.io/shfdb4/.

### Statistical analysis

Temporal trends in SGLT2i prevalent use were presented over the months of inclusion. For each calendar month, prevalent use was calculated as the proportion of patients registered in SwedeHF during that month who had a dispensed prescription of an SGLT2i(numerator), divided by the total number of patients included in that month (denominator). Temporal trends were reported according to sex, age, eGFR (≥60 vs. <60 mL/min/1.73 m^2^), presence of type 2 diabetes mellitus (T2DM), HF hospitalization (HHF) within 1 year, location (in-patient or out-patient), and duration of HF.

Baseline characteristics of SGLT2i users vs. non-users were described by EF, reported as frequencies for categorical variables and median values (quartile 1, quartile 3) for continuous variables, and compared by chi-square and Kruskal-Wallis tests, respectively. Given the registry-based design, EF categories were used to define HF phenotypes among patients with an established clinical diagnosis of HF, in line with common practice in large HF registries. HFrEF was defined as EF <40%, HFmrEF as EF 40–49%, HFpEF as EF ≥50%; the adopted definition of the EF phenotypes was slightly different as compared with the currently used definition since EF was collected as a categorized variable in most cases. Several continuous variables [including systolic blood pressure, heart rate, body mass index, eGFR, and N-terminal pro-b-type natriuretic peptide (NT-proBNP)] were categorized using clinically meaningful cut-offs or tertiles to enhance interpretability and to account for potential non-linear associations with SGLT2i use, in line with the descriptive and implementation-focused aim of the study.

The independent associations between baseline patient characteristics and SGLT2i use were evaluated by a multivariable logistic regression model including the variables shown in *Table 1* and Supplementary material online, *Table S1*, selected based on clinical relevance. Independent predictors were also assessed separately for each EF category, and their potential different role according to EF was evaluated by including an interaction term between relevant patient characteristics and EF category in the multivariable models. Results were reported as odds ratios (ORs) with 95% confidence intervals (CI).

**Table 1:** Baseline characteristics of study population

	HFrEF				HFmrEF				HFpEF			
	Missing (%)	No SGLT2i	SGLT2i	*P*-value	Missing (%)	No SGLT2i	SGLT2i	*P*-value	Missing (%)	No SGLT2i	SGLT2i	*P*-value
*N* (%)		1532 (15)	8998 (85)			1941 (25)	5684 (75)			2342 (36)	4081 (64)	
SGLT2i substance	0			<0.001	0			<0.001	0			<0.001
None		1532 (100)	—			1941 (100)	—			2342 (100)	—	
Dapagliflozin		—	6498 (72)			—	4007 (70)			—	2733 (67)	
Empagliflozin		—	2446 (27)			—	1638 (29)			—	1317 (32)	
Both		—	54 (1)			—	39 (1)			—	31 (1)	
Previous SGLT2i use	0			<0.001	0			<0.001	0			<0.001
No previous use		1118 (73)	<10			1611 (83)	<10			2067 (88)	<10	
Baseline-14 days		—	1423 (16)			—	1035 (18)			—	815 (20)	
4 months-<Baseline		—	3822 (42)			—	1697 (30)			—	925 (23)	
4 months-2 years		248 (16)	2561 (28)			207 (11)	2271 (40)			178 (8)	1893 (46)	
≥2 years		166 (11)	1192 (13)			123 (6)	681 (12)			97 (4)	448 (11)	
Baseline year: quarter^a^	0			<0.001	0			<0.001	0			<0.001
2022:3		83 (5)	292 (3)			116 (6)	134 (2)			113 (5)	79 (2)	
2022:4		220 (14)	771 (9)			265 (14)	466 (8)			309 (13)	290 (7)	
2023:1		187 (12)	844 (9)			275 (14)	563 (10)			304 (13)	385 (9)	
2023:2		175 (11)	884 (10)			206 (11)	515 (9)			246 (11)	417 (10)	
2023:3		140 (9)	749 (8)			172 (9)	523 (9)			239 (10)	356 (9)	
2023:4		149 (10)	928 (10)			205 (11)	644 (11)			248 (11)	468 (11)	
2024:1		188 (12)	1058 (12)			198 (10)	696 (12)			281 (12)	538 (13)	
2024:2		129 (8)	1151 (13)			188 (10)	748 (13)			246 (11)	531 (13)	
2024:3		137 (9)	1080 (12)			147 (8)	647 (11)			178 (8)	451 (11)	
2024:4		124 (8)	1241 (14)			169 (9)	748 (13)			178 (8)	566 (14)	
Sex^a^	0			0.117	0			0.001	0			<0.001
Female		439 (29)	2402 (27)			683 (35)	1756 (31)			1193 (51)	1751 (43)	
Male		1093 (71)	6596 (73)			1258 (65)	3928 (69)			1149 (49)	2330 (57)	
Age (years)	0	75 [66–82]	73 [64–79]	<0.001	0	75 [66–82]	74 [64–80]	<0.001	0	78 [69–83]	77 [68–82]	<0.001
Age (years)^a^	0			<0.001	0			<0.001	0			<0.001
<70		497 (32)	3470 (39)			659 (34)	2074 (36)			621 (27)	1108 (27)	
70–80		575 (38)	3648 (41)			700 (36)	2344 (41)			825 (35)	1683 (41)	
>80		460 (30)	1880 (21)			582 (30)	1266 (22)			896 (38)	1290 (32)	
Location^a^	0			<0.001	0			<0.001	0			<0.001
Out-patient		1297 (85)	8047 (89)			1659 (85)	5233 (92)			1915 (82)	3615 (89)	
In-patient		235 (15)	951 (11)			282 (15)	451 (8)			427 (18)	466 (11)	
Previous HFH <1 year^a^	0	530 (35)	3743 (42)	<0.001	0	441 (23)	1424 (25)	0.042	0	634 (27)	1248 (31)	0.003
Follow-up referral HF nurse clinic^a^	3	1290 (89)	8203 (94)	<0.001	6	1508 (85)	4757 (88)	0.001	11	1539 (75)	2835 (77)	0.101
Follow-up referral speciality^a^	2			<0.001	3			<0.001	6			<0.001
Primary care/Other		288 (19)	862 (10)			562 (30)	1117 (20)			903 (41)	1383 (36)	
Hospital		1191 (81)	7989 (90)			1296 (70)	4443 (80)			1274 (59)	2500 (64)	
Duration of HF (months)^a^	1			<0.001	2			0.078	4			0.121
<6		712 (47)	5021 (56)			895 (47)	2500 (45)			793 (35)	1472 (37)	
≥6		799 (53)	3880 (44)			1004 (53)	3085 (55)			1447 (65)	2462 (63)	
NYHA class	14			0.033	17			0.186	24			0.787
I		172 (14)	1099 (14)			355 (23)	1055 (22)			406 (24)	754 (24)	
II		607 (50)	4207 (54)			789 (52)	2614 (54)			760 (46)	1508 (47)	
III		439 (36)	2506 (32)			375 (25)	1146 (24)			485 (29)	928 (29)	
IV		<10	51 (1)			<10	18 (0)			<10	14 (0)	
NYHA class^a^	14			0.009	17			0.601	24			0.821
I-II		779 (64)	5306 (67)			1144 (75)	3669 (76)			1166 (70)	2262 (71)	
III-IV		445 (36)	2557 (33)			377 (25)	1164 (24)			494 (30)	942 (29)	
BMI (kg/m^2^)	23	26 [23–29]	26 [23–30]	<0.001	31	26 [24–30]	27 [24–30]	0.023	36	26 [24–30]	27 [24–32]	<0.001
BMI (kg/m^2^)^a^	23			<0.001	31			0.072	36			<0.001
<30		912 (80)	5184 (74)			972 (75)	2861 (72)			1056 (73)	1752 (66)	
≥30		226 (20)	1798 (26)			323 (25)	1088 (28)			386 (27)	913 (34)	
Systolic blood pressure (mmHg)	4	124 [110–138]	120 [107–130]	<0.001	5	125 [114–140]	123 [110–136]	<0.001	6	130 [118–144]	126 [115–140]	<0.001
Systolic blood pressure (mmHg)^a^	4			<0.001	5			<0.001	6			<0.001
<140		1113 (76)	7218 (83)			1304 (72)	4191 (77)			1445 (66)	2810 (73)	
≥140		352 (24)	1471 (17)			519 (28)	1218 (23)			737 (34)	1019 (27)	
Diastolic blood pressure (mmHg)	3	74 [66–80]	72 [65–80]	<0.001	5	75 [69–82]	74 [67–80]	<0.001	6	75 [68–80]	73 [67–80]	0.001
Mean arterial pressure (mmHg)	3	91 [82–100]	88 [80–97]	<0.001	5	93 [84–101]	90 [83–99]	<0.001	6	93 [86–101]	91 [83–99]	<0.001
Heart rate (beats/min)	4	72 [64–83]	70 [61–80]	<0.001	6	70 [61–80]	69 [60–78]	<0.001	8	70 [61–80]	69 [60–78]	0.009
Heart rate (beats/min)^a^	4			<0.001	6			<0.001	8			0.031
≤70		684 (47)	4504 (52)			940 (52)	3087 (58)			1141 (54)	2141 (57)	
>70		778 (53)	4161 (48)			866 (48)	2268 (42)			983 (46)	1637 (43)	
eGFR (mL/min/1.73 m2)	0	72 [54–89]	71 [54–87]	0.632	0	75 [56–90]	73 [56–88]	0.015	0	70 [52–87]	66 [51–85]	0.001
eGFR (mL/min/1.73 m2)^a^	0			0.888	0			0.217	0			0.001
≥60		1018 (66)	5999 (67)			1368 (70)	3919 (69)			1507 (64)	2457 (60)	
<60		514 (34)	2999 (33)			573 (30)	1765 (31)			835 (36)	1624 (40)	
Potassium (mmol/L)	1	4.2 [4.0–4.5]	4.3 [4.0–4.5]	0.001	1	4.2 [3.9–4.5]	4.2 [4.0–4.5]	<0.001	1	4.2 [3.9–4.4]	4.2 [4.0–4.5]	<0.001
Potassium (mmol/L)	1			0.245	1			0.525	1			0.060
3.5–5 (Normakalemia)		1435 (95)	8464 (95)			1832 (95)	5398 (96)			2153 (93)	3800 (94)	
<3.5 (Hypokalemia)		44 (3)	206 (2)			48 (2)	117 (2)			103 (4)	135 (3)	
>5 (Hyperkalemia)		39 (3)	270 (3)			44 (2)	136 (2)			59 (3)	117 (3)	
Hemoglobin (g/L)	10	136 [123–149]	141 [129–153]	<0.001	11	135 [122–146]	140 [128–151]	<0.001	9	131 [119–143]	136 [124–149]	<0.001
NT-proBNP (pg/mL)	12	1910 [709–4490]	1687 [685–3920]	0.032	13	952 [315–2271]	915 [328–2174]	0.409	12	986 [329–2327]	1000 [336–2238]	0.777
NT-proBNP (pg/mL)^a^	12			0.036	13			0.597	12			0.648
1st tertile		330 (25)	2058 (26)			703 (42)	2136 (43)			829 (41)	1485 (41)	
2nd tertile		393 (30)	2663 (33)			537 (32)	1627 (33)			677 (33)	1260 (34)	
3rd tertile		576 (44)	3259 (41)			416 (25)	1181 (24)			522 (26)	909 (25)	
Smoking^a^	22	145 (13)	753 (11)	0.013	29	115 (9)	349 (8)	0.546	40	117 (9)	171 (7)	0.007
Diabetes^a^	0	345 (23)	2359 (26)	0.002	0	366 (19)	1381 (24)	<0.001	0	396 (17)	1108 (27)	<0.001
Hypertension^a^	0	1038 (68)	5880 (65)	0.071	0	1315 (68)	3997 (70)	0.036	0	1787 (76)	3198 (78)	0.061
Ischemic heart disease^a^	0	710 (46)	4075 (45)	0.459	0	825 (43)	2530 (45)	0.131	0	844 (36)	1394 (34)	0.135
Stroke^a^	0	228 (15)	1042 (12)	<0.001	0	217 (11)	624 (11)	0.839	0	266 (11)	477 (12)	0.720
Atrial fibrillation/flutter^a^	0	862 (56)	4620 (51)	<0.001	0	972 (50)	3011 (53)	0.029	0	1358 (58)	2605 (64)	<0.001
Anemia^a^	10	406 (30)	1691 (21)	<0.001	11	527 (31)	1052 (21)	<0.001	9	736 (35)	950 (26)	<0.001
Valvular disease^a^	0	277 (18)	1503 (17)	0.196	0	394 (20)	1207 (21)	0.400	0	591 (25)	1010 (25)	0.687
Liver disease^a^	0	58 (4)	234 (3)	0.011	0	41 (2)	119 (2)	1.000	0	68 (3)	102 (2)	0.373
COPD^a^	0	168 (11)	841 (9)	0.052	0	198 (10)	585 (10)	0.943	0	283 (12)	544 (13)	0.162
Malignant cancer^a^	0	246 (16)	1089 (12)	<0.001	0	343 (18)	812 (14)	<0.001	0	418 (18)	640 (16)	0.027
Charlson Comorbidity Index	0	3 [1–4]	2 [1–4]	<0.001	0	2 [1–4]	2 [1–4]	0.001	0	3 [1–4]	3 [1–4]	0.278
Charlson Comorbidity Index	0			<0.001	0			0.007	0			0.345
0–1		392 (26)	2918 (32)			535 (28)	1687 (30)			693 (30)	1215 (30)	
2–3		637 (42)	3568 (40)			799 (41)	2431 (43)			867 (37)	1580 (39)	
4–7		420 (27)	2229 (25)			511 (26)	1353 (24)			664 (28)	1107 (27)	
≥8		83 (5)	283 (3)			96 (5)	213 (4)			118 (5)	179 (4)	
ACEi/ARB/ARNi^a^	0	1383 (90)	8661 (96)	<0.001	0	1646 (85)	5196 (91)	<0.001	0	1702 (73)	3255 (80)	<0.001
Beta-blocker^a^	0	1363 (89)	8464 (94)	<0.001	0	1624 (84)	5072 (89)	<0.001	0	1797 (77)	3434 (84)	<0.001
MRA^a^	0	833 (54)	6708 (75)	<0.001	0	803 (41)	3603 (63)	<0.001	0	898 (38)	2352 (58)	<0.001
Diuretic^a^	0	928 (61)	5530 (61)	0.513	0	925 (48)	2854 (50)	0.056	0	1452 (62)	2665 (65)	0.011
Device therapy^a^	0			0.253	0			0.205	0			0.736
No		1311 (86)	7606 (85)			1810 (93)	5249 (92)			2251 (96)	3922 (96)	
CRT/ICD		218 (14)	1388 (15)			129 (7)	429 (8)			83 (4)	153 (4)	
Family situation^a^	0			0.016	0			0.003	0			0.062
Cohabitating		775 (51)	4869 (54)			1028 (53)	3235 (57)			1164 (50)	2129 (52)	
Living alone		750 (49)	4119 (46)			910 (47)	2444 (43)			1176 (50)	1950 (48)	
Education	1			0.139	1			0.007	1			0.146
Compulsory school		452 (30)	2433 (27)			541 (28)	1386 (25)			664 (29)	1075 (27)	
Secondary school		683 (45)	4155 (47)			852 (44)	2658 (47)			991 (43)	1816 (45)	
University		376 (25)	2279 (26)			525 (27)	1582 (28)			652 (28)	1137 (28)	
Income	0			<0.001	0			<0.001	0			<0.001
1st tertile within year		571 (37)	2957 (33)			667 (34)	1668 (29)			873 (37)	1324 (32)	
2nd tertile within year		543 (36)	2889 (32)			619 (32)	1888 (33)			778 (33)	1367 (34)	
3rd tertile within year		411 (27)	3142 (35)			652 (34)	2123 (37)			689 (29)	1388 (34)	
Socioeconomic gradients^a^	1			<0.001	1			0.001	1			0.001
Low		217 (14)	1084 (12)			262 (14)	591 (11)			347 (15)	472 (12)	
Medium low		681 (45)	3614 (41)			759 (40)	2257 (40)			952 (41)	1675 (42)	
Medium high		440 (29)	2940 (33)			619 (32)	1863 (33)			677 (29)	1247 (31)	
High		173 (11)	1229 (14)			278 (14)	915 (16)			331 (14)	634 (16)	
EQ5D-vas	39	70 [50–80]	70 [50–80]	0.087	47	70 [55–80]	70 [53–80]	0.910	58	70 [50–80]	70 [50–80]	0.521
EQ5D-vas	39			0.074	47			0.673	58			0.200
0–25		46 (5)	209 (4)	0–25		23 (3)	92 (3)			39 (5)	71 (4)	
26–50		217 (26)	1352 (24)	26–50		190 (21)	676 (22)			223 (26)	440 (24)	
51–75		346 (41)	2338 (42)	51–75		390 (43)	1263 (41)			296 (35)	706 (39)	
76–100		239 (28)	1681 (30)	76–100		311 (34)	1072 (35)			297 (35)	602 (33)	

Entries are numbers (percentages) or medians (interquartile ranges).

ACEi, angiotensin-converting enzyme inhibitors; ARB, angiotensin receptor blockers; ARNi, angiotensin receptor/neprilysin inhibitor; BMI, body mass index; CRT/ICD, cardiac resynchronization therapy/implantable cardioverter defibrillator; eGFR, estimated glomerular filtration rate; EQ5D-vas, Euro Quality of Life 5-Dimension questionnaire visual-analogue scale.; HF, heart failure; HFH, HF hospitalization; HFmrEF, HF with mildly reduced ejection fraction; HFpEF, HF with preserved ejection fraction; HFrEF, HF with reduced ejection fraction; MRA, mineralocorticoid receptor antagonist; NT-proBNP, N-terminal prohormone of brain natriuretic peptide; NYHA, New York heart association; SGLT2i, sodium-glucose cotransporter-2 inhibitors.

^a^Included in multiple imputation and regression models.

Outliers were investigated by assessing Cook’s distance, and multicollinearity by analysing the variance inflation factor; no action was deemed necessary. Missing data for variables included in multivariable models were imputed by multiple imputation (*n* = 10) using mice (package 3.18.0), and Rubin’s rules were used for combining estimates and standard errors across imputed data sets. Variables included in the model are indicated in *Table 1*.

All analyses were performed using R version 4.5.1. The level of significance is set to 5%, two-sided.

### Ethics

Individual consent for registration in SwedeHF is not required, but patients are informed of entry into the register and they can opt-out. The establishment of the HF registry and this analysis including the linkage across several registries was approved by the Swedish Ethical Review Authority and complies with the Declaration of Helsinki.

## Results

The overall number of patients was 24,578, of whom 76% were prescribed an SGLT2i and 89% were registered as outpatients. Median age was 75 years (IQR 65–81), 33% were females, 24% had T2DM, 34% had chronic kidney disease (CKD) and 33% had a heart failure hospitalization (HHF) within the last year; 43% had HFrEF, 31% HFmrEF and 26% HFpEF. The use of SGLT2i at the index date was 85% in HFrEF, 75% in HFmrEF and 64% in HFpEF. Supplementary material online, *Figure S1* shows the prevalent use data across different regions in Sweden, further detailed in Supplementary material online, *Table S3*: among patients with HFpEF, SGLT2i use ranged from approximately 38% to over 85% across Swedish regions, with substantial but less pronounced variability also observed in HFrEF and HFmrEF.

### Temporal trends of SGLT2i use according to EF (*Figure 1*)

**Figure 1:**
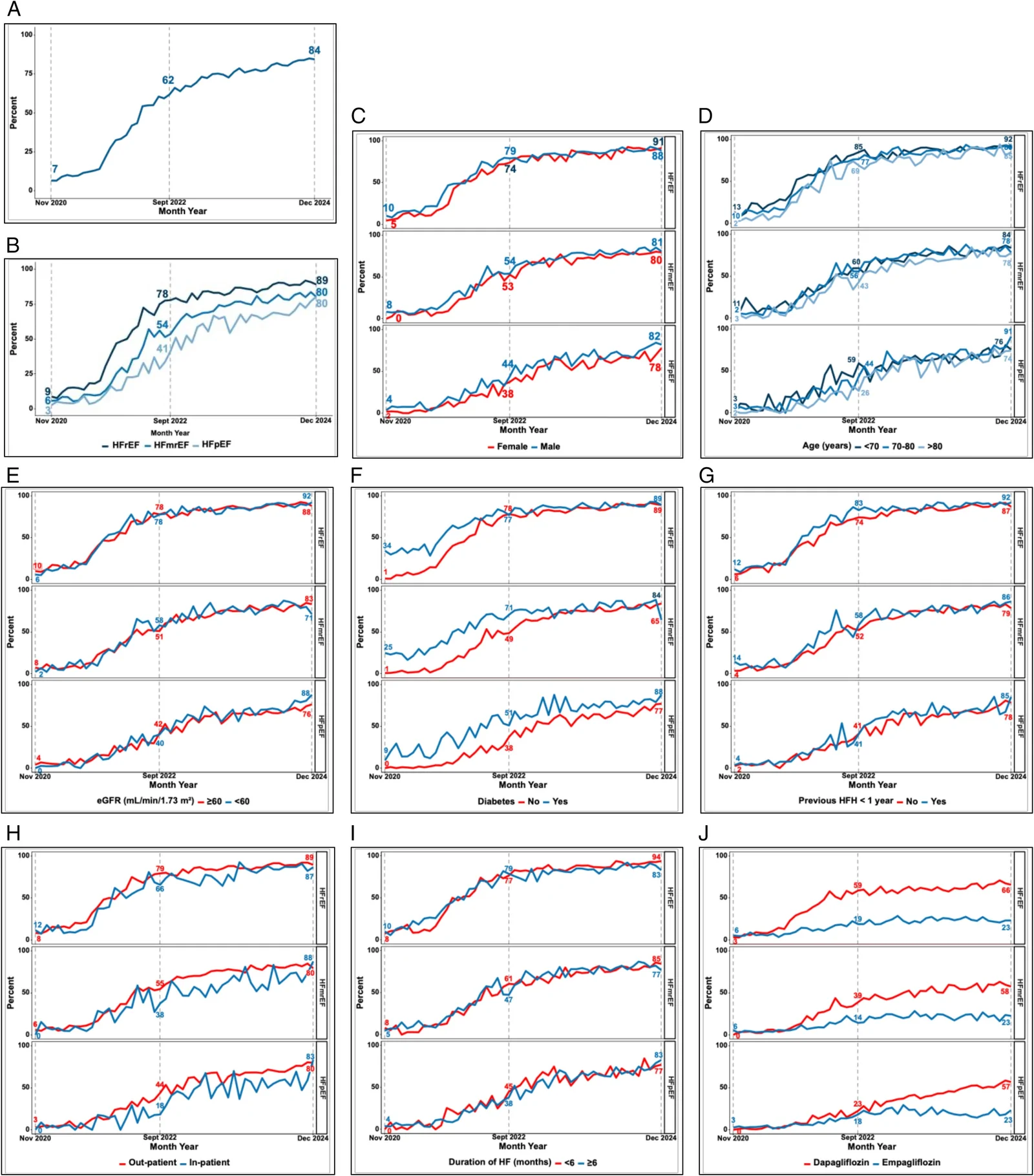
Panels show SGLT2i use over time according to: (*A*) overall population, (*B*) HFrEF vs. HFmrEF vs. HFpEF, (*C*) sex, (*D*) age, (*E*) eGFR, (*F*) diabetes status, (*G*) previous HFH, (*H*) care setting, (*I*) duration of follow-up, and (*J*) dapaglifozin vs. empaglifozin.

In the overall HF population, the use of SGLT2i increased from 7% in November 2020 to 84% in December 2024 (*Figure 1A*); at the end of the study period, the prevalent use was 89% in HFrEF, 80% in HFmrEF and 80% in HFpEF (*Figure 1B*).

Over the study period, SGLT2i use increased substantially in both females and males across all EF categories. In HFrEF, the increase was numerically greater among females (74% to 91%) than males (79% to 88%), whereas similar increases were observed in HFmrEF (53% to 80% vs. 54% to 81%) and HFpEF (39% to 78% vs. 44% to 82%) (*Figure 1C*).

At the end of the study period, treatment use was slightly more prevalent in patients aged <70 years (92% and 84% in HFrEF and HFmrEF, respectively), as compared with those aged 70–80 years (80% and 78% in HFrEF and HFmrEF, respectively) and >80 years (85% and 78% in HFrEF and HFmrEF, respectively). For patients with HFpEF, use was lower in older patients (74% in those aged >80 years, as compared with 76% in <70 and 91% in 70–80), but this group experienced the steepest increase in use over the study period, rising from 25% in 2022 to 74% in 2024 (*Figure 1D*).

SGLT2i use did not vary significantly according to renal function, except for a trend observed during the final months of the study in the HFmrEF group, which eventually resulted in lower use among patients with CKD (71%) as compared with those without CKD (83%) and overall slower uptake over the study period (rise in use from 58% in 2022 to 71% in 2024 in vs. 51% to 83% respectively), suggesting a slower implementation in this subgroup despite overall increasing trends. (*Figure 1E*).

SGLT2i use was higher in patients with T2DM, at the end of the study period, in HFpEF (86% vs. 77% in patients with vs. without T2DM) but not in HFmrEF (65% vs. 84%) (*Figure 1F*).

Overall use of SGLT2i was substantially higher in patients with HHF within the previous year than in those without, regardless of EF (*Figure 1G*).

As regards location of care, at the end of the study period SGLT2i use was higher among inpatients than outpatients in HFmrEF (88% vs. 80%) and HFpEF (83% vs. 80%), with implementation rates between 2022 and 2024 being steeper in inpatients (38% to 88% in HFmrEF and 18% to 83% in HFpEF) as compared with outpatients (55% to 80% in HFmrEF and 44% to 80% in HFpEF) (*Figure 1H*).

There was no difference in SGLT2i use according to HF duration (less or more than 6 months) (*Figure 1I*).

Dapagliflozin was more frequently prescribed than empagliflozin across EF categories (66% vs. 23% in HFrEF, 58% vs. 23% in HFmrEF and 57% vs. 23% in HFpEF; *Figure 1J*).

### Patient characteristics according to SGLT2i use by EF (*Table 1* and Supplementary material online, *Table S1*)

At the index date, in HFrEF, 85% of patients received an SGLT2i, and most non-users had never received the drug (73%). There were no differences in the proportion of females. Users were more frequently in NYHA class II compared to non-users (54% vs. 50%), and had lower NT-proBNP levels (1687 pg/mL [IQR 685–3920 vs. 1910pg/mL (709–4490)]. Users were younger than non-users (73 years [IQR 64–79] vs. 75 years [IQR 66–82]), and more often referred to nurse-led HF clinics (94% vs. 89%) and specialty care (90% vs. 81%). They were also more likely to have experienced a recent (<1 year) HHF and had shorter (<6 months) HF duration. As regards comorbidities, compared with non-users, SGLT2i users were more likely obese (26% vs. 20%) and to have diabetes (26% vs. 23%), but less likely smokers (11% vs. 13%) or with a history of cancer (12% vs. 16%), anaemia (21% vs. 30%), and atrial fibrillation (AF) (51% vs. 56%). The use of renin-angiotensin system inhibitor/angiotensin receptor-neprilysin inhibitor (RASi/ARNi) and beta-blockers (BB) were slightly higher (96% vs. 90% and 94% vs. 89%, respectively), while the use of mineralocorticoid receptor antagonist (MRA) was much higher in SGLT2i users (75% vs. 54%). Users were more likely to have higher socioeconomic gradient. There was no significant difference in quality of life in SGLT2i users vs. non-users.

In HFmrEF, differences between users and non-users were broadly consistent with patterns observed in HFrEF, with some exceptions: users were more likely male than non-users (69% vs. 65%) and no relevant differences were observed in NYHA class or NT-proBNP levels.

In HFpEF, the differences followed a pattern similar to HFmrEF, except for referral to nurse-led HF clinic being comparable in users vs. non-users. Also, users more frequently had impaired renal function (40% vs. 36% in non-users) and a history of AF and were more likely to receive diuretics, oral anticoagulants, and digoxin.

### Independent predictors of use of SGLT2i

**Figure 2:**
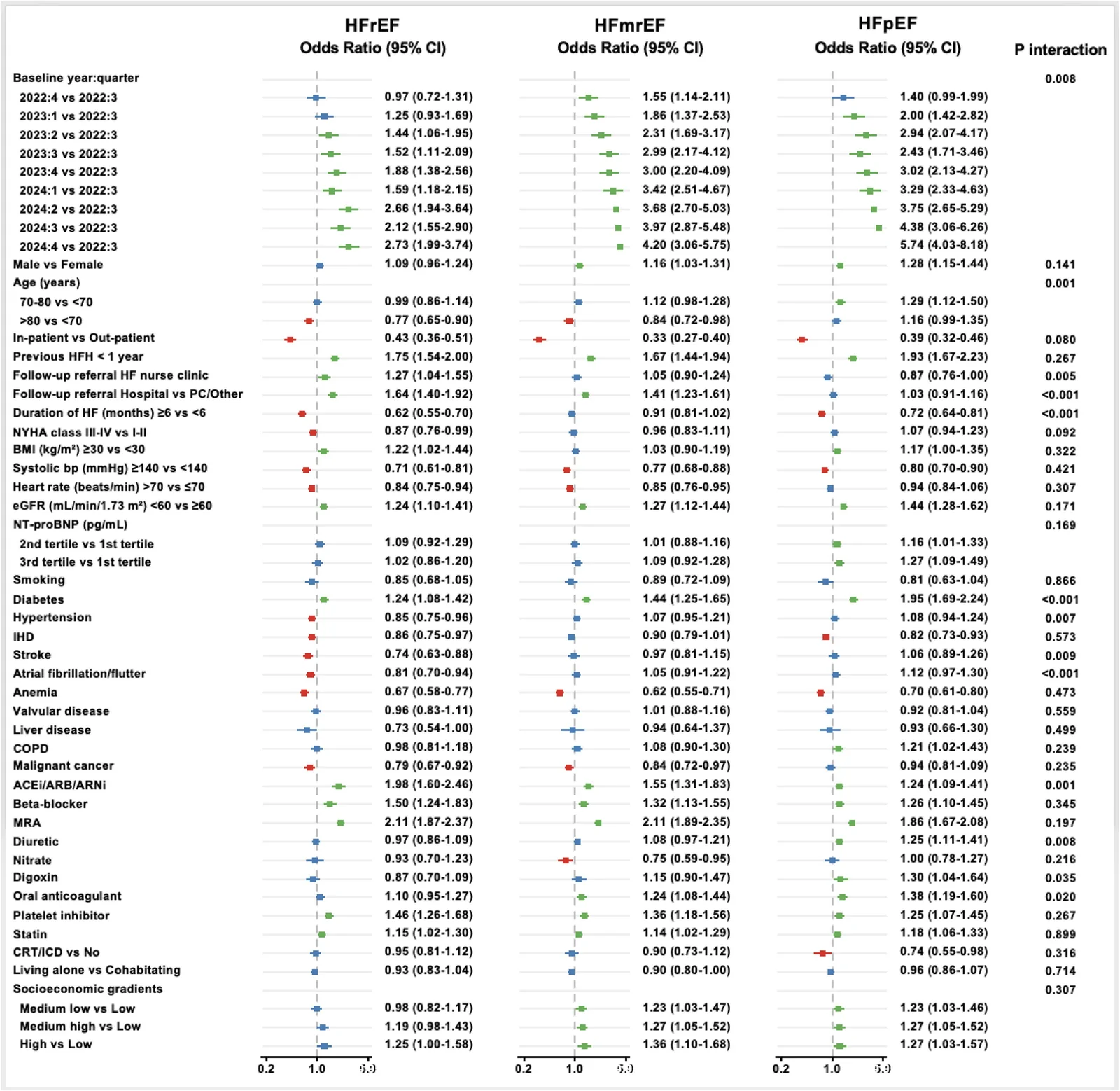
Independent associations between baseline characteristics and SGLT2i, according to ejection fraction. Abbreviations: HFrEF: HF with reduced ejection fraction; HFmrEF: HF with mildly reduced ejection fraction; HFpEF: HF with preserved ejection fraction; SGLT2i: sodium-glucose cotransporter-2 inhibitors; HF: heart failure; HFH: HF hospitalization; NYHA: New York heart association; BMI: body mass index; eGFR: estimated glomerular filtration rate; NT-proBNP: N-terminal prohormone of brain natriuretic peptide; ACEi: angiotensin-converting enzyme inhibitors; ARB: angiotensin receptor blockers; ARNi: angiotensin receptor/neprilysin inhibitor; MRA: mineralocorticoid receptor antagonist; CRT/ICD: cardiac resynchronization therapy/implantable cardioverter defibrillator.

In the overall population, patient characteristics independently associated with higher likelihood of SGLT2i prescription were having HFrEF, a more recent registration in SwedeHF, age 70–80 years old, being registered as out-patient, having had a HHF within the previous year, receiving a follow-up in specialty care, HF duration >6 months, obesity, T2DM, other GDMT use. Impaired kidney function was independently associated with a higher likelihood of SGLT2i use in the overall cohort and across the EF spectrum (HFrEF OR 1.24, 95% CI 1.10–1.41; HFmrEF OR 1.27, 95% CI 1.12–1.44; HFpEF OR 1.44, 95% CI 1.28–1.62), however with a lower uptake over time among HFmrEF patients with CKD. Male sex was independently associated with higher likelihood of SGLT2i use across all EF categories. Although the magnitude of the association was numerically stronger in HFmrEF and HFpEF compared with HFrEF (OR 1.09 (95% CI 0.96–1.24) in HFrEF, OR 1.16 (95% CI 1.03–1.31) in HFmrEF, OR 1.28 (95% CI 1.15–1.44) in HFpEF), no statistically significant sex-by-EF interaction was observed (*P* = 0.141) (*Figure 2*).

In absolute terms, SGLT2i use differed across socioeconomic strata. Patients in the highest socioeconomic gradient had higher SGLT2i use compared with those in the lowest, with similar patterns observed across EF categories and more pronounced differences in HFmrEF and HFpEF.

Age >80 years old, higher blood pressure and heart rate, smoking, ischaemic heart disease, anaemia, history of cancer and living alone were independently associated with a lower likelihood of receiving an SGLT2i (see Supplementary material online, *Figure S2*).

There were also some differences in independent predictors of use according to EF (*Figure 2*). The association between age 70–80 vs. <70 years and SGLT2i use was statistically significant only in HFpEF. Follow-up referral to nurse-led clinics was associated with SGLT2i in HFrEF, and referral to specialty care was an independent predictor of use only in HFrEF and HFmrEF. Shorter (<6 months) HF duration predicted use only in HFrEF and HFpEF. T2DM had a greater association with SGLT2i use in HFpEF than in the other EF groups, despite consistently reaching statistical significance across the EF spectrum. In HFrEF, having AF was associated with lower likelihood of being prescribed with SGT2i. The use of RASi/ARNi was associated with SGLT2i use, and MRA use predicted SGLT2i use at a greater extent in HFrEF and HFmrEF than in HFpEF, however retaining statistical significance across all three groups.

## Discussion

SGLT2i have demonstrated broad effectiveness in HF across the EF spectrum and have a class IA indication in HF treatment across the full EF spectrum in European guidelines on HF.^2–7^ The present analysis highlights important aspects of the trends of SGLT2i use over time, in parallel with their expanded indications to HFmrEF and HFpEF and their increasing uptake in daily practice. Patients with HFmrEF and HFpEF were less likely to receive an SGLT2i compared to HFrEF, consistent with the later trials in these EF categories. However, overall, the implementation of the uptake was remarkably fast, likely due to prior experience with their use in HFrEF, the presence of overlapping indications, their favourable tolerability profile, and the fact that they were the first drugs to be approved for use in HFpEF. Nonetheless, there are still subgroups of patients where the use of these drugs can be better implemented. Importantly, the implementation of SGLT2i should be interpreted within the broader and well-recognized challenge of therapy optimization in HF. Recent implementation-focused analyses have demonstrated that, despite strong guideline recommendations and robust evidence, the translation of GDMT into routine clinical practice remains incomplete. Barriers to optimal implementation operate at multiple levels, including patient-related factors (such as advanced age, multimorbidity, and frailty), provider-related factors (therapeutic inertia and risk perception in complex patients), and system-level factors (care fragmentation, limited access to specialist services, and heterogeneity in healthcare organization). These challenges are particularly pronounced in older, multimorbid, and socially vulnerable populations—subgroups in whom we also observed lower SGLT2i uptake in the present study.^15,16^

### Temporal trends in SGLT2i use and regional differences

SGLT2i use increased by 17% from September 2022 to December 2024, more in HFmrEF (from 54% to 80%) and HFpEF (41% to 80%) than in HFrEF (78% to 89%). The implementation in HFrEF is probably reaching a plateau, consistent with trends observed for RASi and BB use, which has been stable around 90% in Sweden over the past decades.^17^ The steep increase in SGLT2i use in HFmrEF and HFpEF reflects the publication of the DELIVER trial in September 2022, showing that dapagliflozin reduced the relative risk of a composite of worsening HF/cardiovascular death by 18%, and confirming the earlier results of the EMPEROR-Preserved trial, where empagliflozin reduced the risk of HHF/cardiovascular death by 21%, and the subsequent update of ESC Guidelines on HF with a class IA recommendation for SGLT2i in HFmrEF/HFpEF in August 2023. Interestingly, we report an SGLT2i use up to 20–25% among HFmrEF and HFpEF patients without diabetes even before the approval of empagliflozin by the European Medical Agency in January 2022 and by the Swedish Medical Products Agency in April 2022, suggesting that clinicians might have started to prescribe SGLT2i off-label following the divulgation of trial data.^18^ The present finding of almost 90% of patients registered in SwedeHF being prescribed an SGLT2i by the end of 2024, with rates of 80% in HFmrEF and HFpEF only few months after guidelines update and two years after label update (January 2022 for empagliflozin and December 2022 for dapagliflozin),^18,19^ suggests good dissemination of knowledge, proper implementation of trials findings and guidelines. This is likely facilitated by physicians’ familiarity with this pharmacological class already used in HFrEF, overlapping indications with T2DM and CKD, as well as their favourable safety profile and convenience of use.

However, some aspects require further consideration. SGLT2i use increased substantially in both females and males across EF categories, although women consistently showed slightly lower absolute use than men, particularly in HFmrEF and HFpEF, in line with previous reports of SGLT2i’s more limited use in females regardless of the indication.^20,21^ While concerns regarding genital and urinary tract infections may contribute to prescribing hesitancy, additional mechanisms are likely involved. Women with HFpEF often present with greater phenotypic heterogeneity, higher prevalence of obesity and AF, and less overt congestion, which may lead to diagnostic uncertainty and delayed initiation of disease-modifying therapies.^22^ SGLT2i use tended to be higher in patients with T2DM, particularly in HFpEF, likely reflecting the existence of a prior indication for these drugs in T2DM and the greater likelihood of treatment implementation when multiple indications coexist.

The trend in initiation of SGLT2i was steeper in patients with a HHF in the previous year but also in the inpatient vs. outpatient setting, particularly in HFmrEF and HFpEF, in line with results from a European survey and with evidence from the STRONG-HF trial that rapid initiation and up-titration of GDMT after admission for HF is effective and well-accepted (although treatment in STRONG-HF did not include SGLT2i).^23,24^ Slight age discrepancies were reported, with lower prescription rates in older patients with HFpEF. This may reflect a more cautious approach by prescribers when initiating SGLT2 inhibitors in patients with multiple comorbidities, where polypharmacy and less intensive follow-up are common concerns. However, a trend in increase in use over the study period was observed even in patients aged >80 years, reflecting the well-received safety profile of these drugs.^10^

Previous work reports that, in patients with CKD, the uptake of SGLT2i has been slow despite the demonstrated favourable effects on kidney outcomes and other outcomes in this subgroup.^25,26^ The low use of SGLT2i in patients with CKD and T2DM up to 2020 has been previously reported in analyses from the Center for Kidney Disease Research, Education and Hope (CURE-CKD) registry (6%) and the Veterans Health Administration system (11%).^27,28^ In the Get With The Guidelines-Heart Failure (GWTG-HF) registry, including patients with HFrEF, it was reported that SGLT2i prescription was less likely among patients with CKD compared with those with normal renal function (19% vs. 22%).^29^ In 69 680 patients hospitalized for HF 2019–2022 from the Veterans Affairs Healthcare System, CKD was not associated with higher use of SGLT2i at discharge, in line with our previous report from SwedeHF.^9,30^ The underuse of SGLT2i in CKD patients is noteworthy, especially since this condition has an independent indication for this class of drugs, and may be attributed to persistent concerns about the transitory decline in eGFR due to an initial increase in diuresis. By contrast, in the overall cohort of our study, impaired kidney function was independently associated with a higher likelihood of SGLT2i use. In EF-specific models, CKD was associated with SGLT2i use across the whole spectrum of EF, indicating that renal dysfunction *per se* did not represent a barrier to prescription in contemporary Swedish HF care. However, temporal trend analyses revealed a slower uptake and lower absolute use toward the end of follow-up among HFmrEF patients with CKD compared with those without CKD. This suggests that residual hesitancy may persist in specific clinical contexts, particularly in HFmrEF, where therapeutic uncertainty and heterogeneous patient profiles may complicate treatment decisions. Importantly, the CKD population in SwedeHF predominantly comprises patients with mild-to-moderate renal impairment, as patients with eGFR <25 mL/min/1.73m^2^ were excluded. This differs from cohorts such as CURE-CKD and Veterans Affairs analyses, which included patients with more advanced kidney disease, and may partly explain discrepancies across studies.^27,30^

We also report differences in SGLT2i use in different Swedish regions, with Southern regions having higher initiation rates compared with Northern regions, especially as regards HFpEF. It has to be considered that Southern Sweden is substantially more populated than Northern Sweden and has a higher number of hospitals and that the registration in SwedeHF, although quite well-distributed, is still uneven. Therefore, it is possible that the picture does not fully reflect the practice of a representative number of centres per region. Beyond differences in population density and registry coverage, such regional variation may also reflect structural factors, including access to specialist HF care, organization of outpatient services, and urban–rural differences.

### Patient characteristics independently associated with use of SGLT2i

Quantifying use is helpful as a general quality measure, but assessing underlying factors independently associated with use (and non-use) offers information that is more addressable. More recent registration in SwedeHF, a HHF within the past year, impaired kidney function and T2DM were independent predictors of SGLT2i use across EF categories. This generally reflects the settings of implementation discussed when analysing trends. Anaemia and cancer were associated with a lower likelihood of receiving an SGLT2i. Both conditions may be considered proxies for frailty, thus indicating that SGLT2i are less used in patients with multiple comorbidities and decreased functional status, despite the positive effects of SGLT2i on haemoglobin levels, haematocrit and iron metabolism.^31–33^ The use of other classes of GDMT was associated with SGLT2i use, possibly underlining that setting and quality of care are pivotal aspects when interpreting real-world findings in implementation science. Accordingly, higher socioeconomic status, education and cohabitation were all positively associated with SGLT2i use, as extensively reported in previous studies by this group.^9,34^ These findings indicate clear socioeconomic gradients in SGLT2i implementation, suggesting that access to and uptake of evidence-based HF therapies remain socially patterned even within a universal healthcare system.

When considering predictors of use according to EF, some important differences emerged. Follow-up referral to a nurse-led clinic was independently associated with a higher likelihood of being prescribed SGLT2i in patients with HFrEF but not in those with HFmrEF and HFpEF. This finding aligns with prior SwedeHF data and may reflect more frequent referral of patients with HFrEF to nurse-led clinics, driven by greater need for initiation and up-titration of GDMT compared with HFmrEF and HFpEF, which have fewer Class I options.^34^ This is also corroborated by our finding that SGLT2i use was associated with referral to specialty care vs. primary care in HFrEF and HFmrEF, but not in HFpEF. Interestingly, the presence of device-based therapy was not strongly associated with higher SGLT2i use, suggesting that access to advanced HF interventions does not necessarily translate into optimal implementation of newer disease-modifying pharmacological therapies. Also, in the group of patients with HFrEF, AF emerged as associated with a lower likelihood of receiving an SGLT2i, this being in line with an analysis of American insurance data reporting on patients with comorbid HFrEF and AF.^35^ The reason for this association is unclear, but physicians might judge dyspnoea and symptoms more related to AF than to HF, and therefore prioritize treatments for the first.

### Incremental contribution compared with previous SwedeHF analyses

Compared with our previous SwedeHF analysis restricted to HFrEF, the present study provides several novel insights. First, it extends the evaluation of SGLT2i implementation to HFmrEF and HFpEF, allowing assessment of uptake following randomized evidence and Class IA recommendations across the full EF spectrum. Second, the extended observation window captures the period after publication of DELIVER and EMPEROR-Preserved and the 2023 ESC focused update, thus reflecting implementation in a fully contemporary therapeutic context. Third, we demonstrate EF-specific prescription patterns and disparities, including more pronounced sex differences and stronger influence of diabetes in HFpEF, as well as socioeconomic and regional gradients in use. These findings suggest that determinants of implementation are not uniform across EF phenotypes and highlight the need for tailored strategies to optimize therapy delivery.

### Limitations

Because of the observational nature of the study, the presence of residual confounders cannot be ruled out. The generalizability of our results to the whole of Sweden is somewhat limited by the incomplete coverage in SwedeHF, potentially leading to an overestimation of SGLT2i use, since patients enrolled in the registry are generally better treated compared with the overall Swedish HF population.^36^

In addition, SGLT2i use was assessed based on dispensed medications, which represents an approximation of treatment implementation. This approach does not capture in-hospital use, prescriptions that were not filled, or short-term discontinuation after prescription.

Furthermore, given the observational design and the registry-based setting, indication and selection bias cannot be excluded. Patients with more severe HF, higher comorbidity burden, or more structured follow-up may be both more likely to receive SGLT2i and to be enrolled in SwedeHF, potentially leading to an overestimation of SGLT2i implementation compared with the overall national HF population. The exposure window for SGLT2i was chosen considering the three months duration of prescriptions in Sweden, to be sure to capture dispensations; we did not perform any sensitivity analyses using different intervals, which may be considered in future work.

Finally, the categorization of continuous variables may have led to some loss of information and statistical power; however, this approach was chosen to facilitate clinical interpretability in the context of an implementation-focused analysis. Specifically, given the registry-based design, EF categories were used to define HF phenotypes in line with common practice; however, the use of categorical EF ranges may have attenuated differences across EF strata, particularly near EF boundaries.

### Conclusions

There has been a remarkably rapid implementation of SGLT2i uptake in SwedeHF, a generalizable registry reflecting clinical practice. Despite the fast implementation, certain disparities persist, particularly in female patients, older individuals, and those with lower socioeconomic status. Regional variations in prescription patterns highlight potential healthcare access and implementation gaps. While the overall trend shows strong physicians’ adherence to evidence-based practices, further efforts are needed to ensure equitable and comprehensive adoption of SGLT2i therapy across all eligible patient populations, particularly those with HFpEF.

## Supplementary Material

qcag063_Supplementary_Data

## Data Availability

The data underlying this article will be shared on reasonable request to the corresponding author.
