# Supplementary material for: Implementation of sodium-glucose co-transporter 2 inhibitors use in patients with heart failure across the ejection fraction: data from the Swedish heart failure registry
Source: Eur Heart J Qual Care Clin Outcomes. 2026 Apr 19;12(6):947–61. doi: 10.1093/ehjqcco/qcag063 (PMC13579327; doi:10.1093/ehjqcco/qcag063)
Supplement: Supplementary file 1 [file qcag063_supplementary_data.docx]

**Supplemental Table 1**. Flowchart and inclusion/exclusion criteria of the study population.

| **General inclusion/exclusion criteria** | |
| --- | --- |
| No of posts recieved | 270768 |
| Exclude posts with duplicated index dates | 270145 |
| Exclude posts with index date < 2000-01-01 | 270142 |
| Exclude posts with index date > 2024-12-31 | 264618 |
| Exclude posts < 18 years | 264602 |
| Exclude posts with reused or incorrect PINs | 264302 |
| Exclude posts end of follow-up < index | 264007 |
| Exclude posts with end of follow-up <= index date (died in hospital) | 260668 |
| **Project-specific inclusion/exclusion criteria** | |
| Include posts >= 2020-11-01 | 74680 |
| Exclude post with missing EF | 54698 |
| Exclude posts with missing DM type | 53772 |
| Exclude posts with DM type I | 53109 |
| Exclude posts with missing eGFR | 50334 |
| Exclude posts with eGFR < 25 | 49362 |
| Exclude posts with previous dialysis within 5 years | 49092 |
| Exclude posts with <= 14 days follow-time (SGLT2i defined as dispensations up untill 14 days after baseline) | 48648 |
| Last post / patient (Used for temporal trends) | 37012 |
| Include posts >= 2022-09-01 (used for all other analyses) | 24578 |

**Legend**

PIN: personal identification number; EF: ejection fraction; DM: diabetes mellitus; eGFR: estimated glomerular filtration rate; SCB: SGLT2i: sodium-glucose co-transporter 2 inhibitors.

**Supplemental Table 2**. Variable definition.

| **Variable** | **Data source** |
| --- | --- |
| SGLT2i | NPDR: ATC A10BK01, A10BD15, A10BD21, A10BD25, A10BK03, A10BD19, A10BD20 ^c^ |
| Empagliflozin | NPDR: ATC A10BK03, A10BD19, A10BD20 ^c^ |
| Dapagliflozin | NPDR: ATC A10BK01, A10BD15, A10BD21, A10BD25 ^c^ |
| Baseline year:quarter | SwedeHF |
| Sex | SwedeHF |
| Age | SwedeHF |
| Location | SwedeHF |
| Previous HFH < 1 year | SwedeHF; NPR |
| Follow-up referral HF nurse clinic | SwedeHF |
| Follow-up referral speciality | SwedeHF |
| Duration of HF | SwedeHF |
| NYHA class | SwedeHF |
| BMI | SwedeHF |
| Systolic blood pressure | SwedeHF |
| Diastolic blood pressure | SwedeHF |
| Mean arterial pressure | SwedeHF |
| Heart rate | SwedeHF |
| eGFR | SwedeHF CKD-EPI 2021 |
| Potassium | SwedeHF |
| Hemoglobin | SwedeHF |
| NT-proBNP | SwedeHF |
| Smoking | SwedeHF |
| Diabetes | SwedeHF; NPR: ICD-10 E10-4 ^a^ |
| Hypertension | SwedeHF; NPR: ICD-10 I10-5 ^a^ |
| Ischemic heart disease | SwedeHF; NPR: ICD-9/10 410-4, I20-5, Z951, Z955 Procedure FNG, FNA, FNB, FNC, FND, FNE, FNF, FNH ^a^ |
| Stroke | NPR: ICD-9/10 430-4, 438, I60-4, I690-4 ^a^ |
| Atrial fibrillation/flutter | SwedeHF; NPR: ICD-10 I48 ^a^ |
| Anemia | SwedeHF Female Hb < 120 g/L/Male Hb < 130 g/L |
| Valvular disease | NPR: ICD-10 I05-8, I34-9, Q22, Q230-3, Q230-3, Q235-9, Z952-4 ^a^ |
| Liver disease | NPR: ICD-10 B18, I85, I864, I982, K70, K710, K711, K713-7, K72-4, K760, K762-9 ^a^ |
| COPD | NPR: ICD-10 J40-4 ^a^ |
| Malignant cancer | NPR: ICD-10 C ^a^ |
| Charlson Comorbidity Index | NPR ^b^ |
| ACEi/ARB/ARNi | SwedeHF |
| Beta-blocker | SwedeHF |
| MRA | SwedeHF |
| Diuretic | SwedeHF |
| Nitrate | SwedeHF |
| Digoxin | SwedeHF |
| Oral anticoagulant | SwedeHF |
| Platelet inhibitor | SwedeHF |
| Statin | SwedeHF |
| Device therapy | SwedeHF |
| Family situation | LISA |
| Education | LISA |
| Disposable income | LISA |
| Socioeconomic gradients | LISA |
| EQ5D-vas | SwedeHF |

^a^ Derived from diagnoses/operations in all positions, in-patient and out-patient speciality care within 5 years prior to index (ICD-10)/1987 (ICD-9) unless stated otherwise.

^b^ Derived from diagnoses in all positions, in-patient and out-patient speciality care within 10 years prior to index using ICD-9/10 according to Ludvigsson, 2021 (see reference).

^c^ Medications derived from dispensed prescriptions within 4 months prior to baseline up untill 14 days after.

**Legend**

HF: heart failure; SedeHF: Swedish HF registry; NPR: National Patient Registry; NYHA: New York Heart Association; BMI: body mass index; CKD-EPI: Chronic Kidney Disease Epidemiology Collaboration; eGFR: estimated glomerular filtration rate; NT-proBNP: N-terminal prohormone of brain natriuretic peptide; ICD: international collaboration of diseases: COPD: chronic obstructive pulmonary disease; SGLT2i: sodium-glucose co-transporter 2 inhibitors; NPDR: National Prescribed Drug Registry; ACEi: angiotensin-converting enzyme inhibitors; ARB: angiotensin receptor blockers; ARNi: angiotensin receptor/neprilysin inhibitor; MRA: mineralocorticoid receptor antagonist; LISA: Longitudinal integrated database for health insurance and labour market studies.

**Supplemental Table 3**. SGLT2i use at registration in SwedeHF by county of centre (number, percentage).

| **County** | **HFrEF** | **HFmrEF** | **HFpEF** |
| --- | --- | --- | --- |
| Blekinge | 266 (94.3) | 107 (90.7) | 36 (85.7) |
| Dalarna | 228 (83.8) | 107 (73.3) | 54 (71.1) |
| Gävleborg | 280 (91.2) | 121 (78.1) | 61 (82.4) |
| Gotland | 64 (80.0) | 68 (81.0) | 59 (53.6) |
| Halland | 244 (87.1) | 79 (69.9) | 97 (57.7) |
| Jämtland | 94 (90.4) | 18 (90.0) | 12 (92.3) |
| Jönköping | 355 (78.9) | 294 (68.9) | 190 (54.0) |
| Kalmar | 74 (83.1) | 50 (87.7) | 19 (55.9) |
| Kronoberg | 101 (91.0) | 18 (81.8) | <10 |
| Norrbotten | 168 (79.6) | 98 (62.8) | 33 (49.3) |
| Orebro | 283 (84.2) | 236 (78.4) | 257 (63.9) |
| Östergötland | 638 (86.4) | 316 (72.6) | 139 (60.4) |
| Skåne | 787 (87.0) | 408 (73.6) | 227 (59.7) |
| Södermanland | 354 (85.5) | 214 (65.6) | 170 (55.7) |
| Stockholm | 2175 (84.4) | 1972 (75.3) | 1701 (66.5) |
| Uppsala | 345 (85.4) | 231 (76.2) | 274 (63.1) |
| Värmland | 277 (89.9) | 202 (90.2) | 159 (90.3) |
| Västerbotten | 204 (76.7) | 133 (68.2) | 39 (50.0) |
| Västernorrland | 322 (78.7) | 188 (65.7) | 92 (57.1) |
| Västmanland | 220 (89.1) | 135 (76.3) | 65 (54.2) |
| Västra Götaland | 1519 (87.2) | 689 (76.0) | 391 (61.8) |

**Legend**

SGLT2i: sodium-glucose cotransporter 2 inhibitor; SwedeHF: Swedish Heart Failure Registry; HFrEF: HF with reduced ejection fraction; HFmrEF: HF with mildly reduced ejection fraction; HFpEF: HF with preserved ejection fraction.

**Supplemental Table 4**. Concomitant therapies.

|  | **HFrEF** | | | | **HFmrEF** | | | | **HFpEF** | | | |
| --- | --- | --- | --- | --- | --- | --- | --- | --- | --- | --- | --- | --- |
|  | Missing (%) | No SGLT2i | SGLT2i | p-value | Missing (%) | No SGLT2i | SGLT2i | p-value | Missing (%) | No SGLT2i | SGLT2i | p-value |
| **N (%)** |  | 1532 (15) | 8998 (85) |  |  | 1532 (15) | 8998 (85) |  |  | 1532 (15) | 8998 (85) |  |
| **Nitrate ^a^** | 0 | 68 (4) | 354 (4) | 0.391 | 0 | 121 (6) | 271 (5) | 0.013 | 0 | 137 (6) | 211 (5) | 0.270 |
| **Digoxin ^a^** | 0 | 113 (7) | 605 (7) | 0.379 | 0 | 102 (5) | 339 (6) | 0.274 | 0 | 133 (6) | 307 (8) | 0.006 |
| **Oral anticoagulant ^a^** | 0 | 845 (55) | 4856 (54) | 0.411 | 0 | 984 (51) | 3034 (53) | 0.047 | 0 | 1333 (57) | 2581 (63) | <0.001 |
| **Platelet inhibitor ^a^** | 0 | 393 (26) | 2687 (30) | 0.001 | 0 | 494 (25) | 1631 (29) | 0.007 | 0 | 437 (19) | 782 (19) | 0.661 |
| **Statin ^a^** | 0 | 783 (51) | 5137 (57) | <0.001 | 0 | 998 (51) | 3287 (58) | <0.001 | 0 | 1061 (45) | 2134 (52) | <0.001 |

^a^ Included in multiple imputation and regression models.

**Legend**

SGLT2i: sodium-glucose cotransporter-2 inhibitors; HFrEF: heart failure with reduced ejection fraction; HFmrEF: heart failure with mildly reduced ejection fraction; HFpEF: heart failure with preserved ejection fraction.

**Supplemental Figures**

**Supplemental Figure 1**. SGLT2i use at registration in SwedeHF by county of centre.

**Legend**

SGLT2i: sodium-glucose cotransporter 2 inhibitor; SwedeHF: Swedish Heart Failure Registry; HFrEF: HF with reduced ejection fraction; HFmrEF: HF with mildly reduced ejection fraction; HFpEF: HF with preserved ejection fraction.

**Supplemental Figure 2**. Independent predictors of use of SGLT2i in the whole population.

**Legend**

SGLT2i: sodium-glucose co-transporter 2 inhibitors; LVEF: left ventricular ejection fraction; CI: confidence interval; HFrEF: HF with reduced ejection fraction; HFmrEF: HF with mildly reduced ejection fraction; HFpEF: HF with preserved ejection fraction; SGLT2i: sodium-glucose cotransporter-2 inhibitors; HF: heart failure; HFH: HF hospitalization; NYHA: New York heart association; BMI: body mass index; eGFR: estimated glomerular filtration rate; NT-proBNP: N-terminal prohormone of brain natriuretic peptide; IHD: ischemic heart disease; ACEi: angiotensin-converting enzyme inhibitors; ARB: angiotensin receptor blockers; ARNi: angiotensin receptor/neprilysin inhibitor; MRA: mineralocorticoid receptor antagonist; CRT/ICD: cardiac resynchronization therapy/implantable cardioverter defibrillator.
